# Rheumatic diseases and latent tuberculosis infection in children: diagnostic and therapeutic challenges

**DOI:** 10.3389/fcimb.2025.1659211

**Published:** 2025-09-26

**Authors:** Chenxi Li, Xiangyuan Chen, Ping Zeng

**Affiliations:** Department of Allergy, Immunology and Rheumatology, Guangzhou Women and Children’s Medical Center, Guangzhou Medical University, Guangdong Provincial Clinical Research Center for Child Health, Guangzhou, China

**Keywords:** rheumatic diseases, children, latent tuberculosis infection, diagnosis, prophylactic treatment

## Abstract

Latent tuberculosis infection (LTBI) is a state of sustained immune response to Mycobacterium tuberculosis (MTB) antigens, without clinical evidence of active tuberculosis. Rheumatic diseases, a common type of autoimmune disease, are often treated with glucocorticoids, immunosuppressants, biologics, and small-molecule targeted drugs. These medications can cause immune dysfunction in patients, increasing the risk of latent tuberculosis reactivation. Children with rheumatic diseases are particularly susceptible to MTB due to their immature immune systems, the nature of their rheumatic disease, and the use of anti-rheumatic medications. This susceptibility makes LTBI more likely to progress to active tuberculosis. Therefore, it is crucial to prioritize LTBI screening in children with rheumatic diseases, identify LTBI promptly, and initiate preventive antituberculosis treatment to prevent the onset of active tuberculosis and ensure the health of children with rheumatic diseases. This article discusses the susceptibility mechanisms, diagnostic methods, and preventive antituberculosis treatment strategies for children with rheumatic diseases and LTBI, aiming to reduce the risk of progression to active tuberculosis and improve patient outcomes.

## 1 Introduction

According to the World Health Organization (WHO) 2024 Global Tuberculosis Report, in 2023, of the globally estimated 10.8 million incident tuberculosis cases, children and young adolescents aged 0–14 years accounted for 12%, equivalent to approximately 1.3 million cases. In terms of mortality, among HIV-negative individuals who died from tuberculosis in 2023, 15% (around 166,000) were children and young adolescents under 15 years of age; among those with HIV, this age group constituted 16% (about 25,000) of tuberculosis-related deaths (Global tuberculosis report, 2024).

Mycobacterium tuberculosis, the bacterium that causes TB, is the leading infectious killer globally, accounting for the highest death toll among transmissible diseases and a major contributor to fatalities linked to antimicrobial resistance (Chai et al., 2025). MTB is transmitted via aerosols and initially infects lung macrophages, where it replicates before spreading to lymph nodes and disseminating systemically. The host immune response forms granulomas to contain the infection, leading to latent TB in most cases (Lin and Flynn, 2010). LTBI is a persistent immune response to MTB antigens, with no clinical evidence of active tuberculosis identified through imaging and symptomatic examinations (Shah and Dorman, 2021). Although LTBI is not contagious, approximately 5% to 15% of LTBI cases may progress to active tuberculosis. Therefore, preventing LTBI from progressing to tuberculosis is an important measure for achieving both individual and public health objectives (Shah and Dorman, 2021).

Rheumatic diseases constitute a heterogeneous group of disorders characterized by musculoskeletal system alterations and systemic manifestations; this group includes autoimmune connective tissue diseases, autoinflammatory diseases, and vasculitides. Patients with rheumatic diseases exhibit a higher incidence of tuberculosis than the general population—a risk elevated by two- to tenfold in adults. This increased susceptibility is associated with the immunosuppressive effects of the underlying disease pathology, as well as with therapeutic immunosuppressive agents, such as corticosteroids and biologic drugs (Lima et al., 2023). Telefon et al. carried out a retrospective study to examine how the use of biologic agents affects the occurrence of tuberculosis in kids suffering from chronic inflammatory conditions (Telefon et al., 2025). The majority of patients monitored were those with juvenile idiopathic arthritis (JIA), accounting for 191 cases, or 70.7% of the total. While the emergence and growing application of biologic agents have significantly improved the management of pediatric rheumatic diseases, such therapies also come with an inherent risk of tuberculosis reactivation or development. Moreover, rheumatic children under 5 years of age with LTBI are more susceptible to progression to severe tuberculosis (Chai et al., 2025).

Currently, evidence-based consensus guidelines for the diagnosis and treatment of LTBI in adult patients with rheumatic diseases have been established internationally. However, there are few studies on pediatric rheumatic diseases complicated by LTBI. This paper therefore aims to synthesize the latest advances in screening, diagnosis, and treatment strategies for LTBI specifically within the context of pediatric rheumatology. The ultimate goal is to empower clinicians to proactively identify and manage LTBI through targeted screening and timely initiation of preventive therapy. Such a strategy is imperative to mitigate the risk of active tuberculosis, thereby safeguarding this high-risk cohort and ensuring the safe continuation of essential immunosuppressive treatments for their underlying rheumatic conditions.

## 2 Results

**Figure 1:**
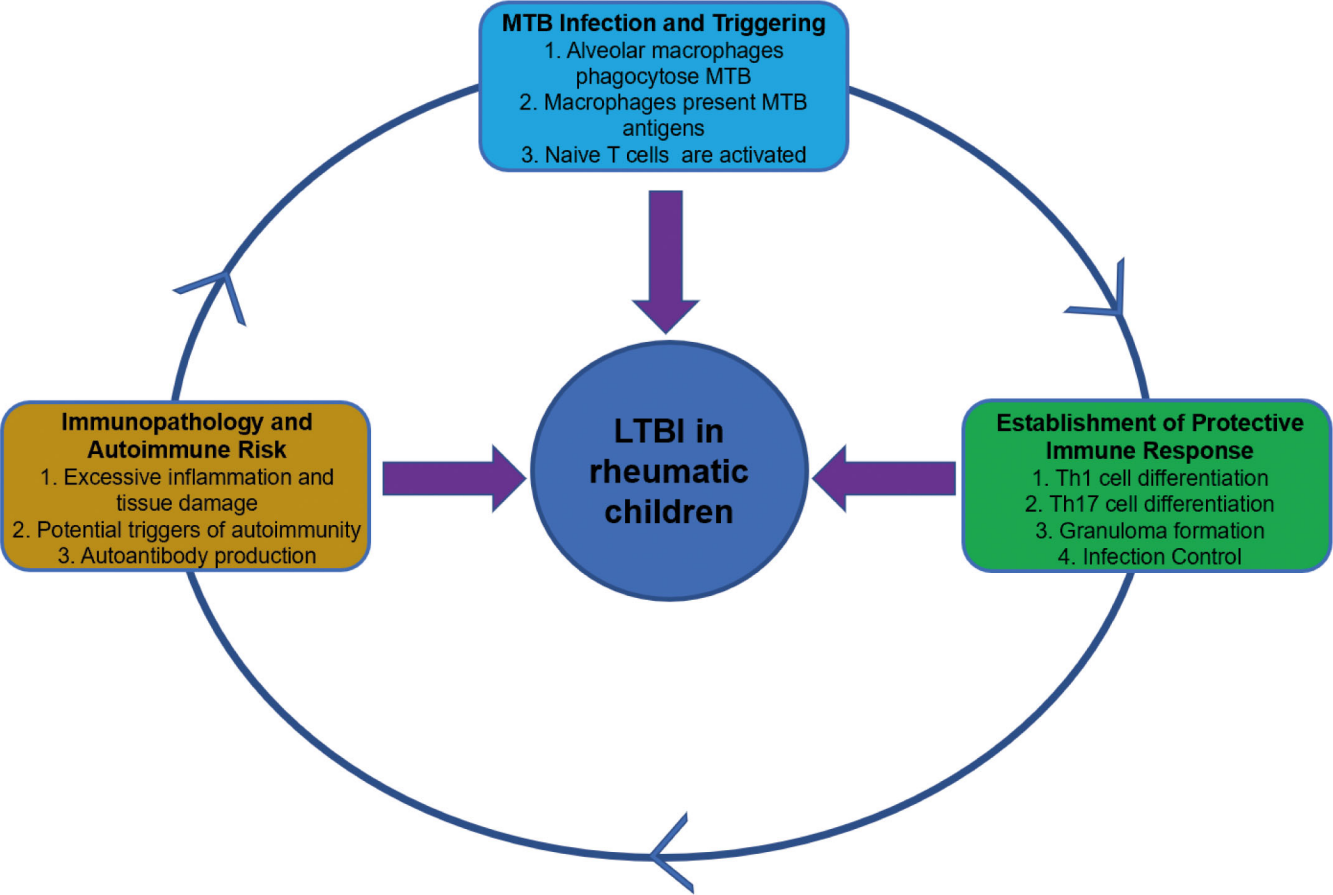
The immune response triggered by MTB infection and its potential autoimmune consequences.

Rheumatic diseases are a heterogeneous group of disorders characterized by musculoskeletal alterations and systemic manifestations. Due to underlying immune dysregulation and the inherent immunosuppression caused by first-line therapies such as corticosteroids, conventional disease-modifying antirheumatic drugs (DMARDs), and biologic agents, patients with rheumatic diseases are at a significantly increased risk of developing tuberculosis. The association between rheumatic diseases and tuberculosis is well-established epidemiologically, with studies indicating that the incidence of tuberculosis among patients with rheumatic diseases is 2 to 10 times higher than that in the general population. **Figure 1** illustrates how the immune cells drive the pathogenesis of tuberculosis and may ultimately lead to autoimmune diseases. In short, MTB infection elicits a Th1/Th17-driven immune response that forms granulomas to contain the pathogen. However, excessive inflammation and antigenic mimicry can break down immune tolerance, triggering autoimmunity. This process exacerbates tissue damage and worsens clinical outcomes (Churilov et al., 2024; Jha et al., 2025; Picchianti-Diamanti et al., 2025).

### 2.1 The pathogenesis of LTBI susceptibility in children with rheumatic diseases

Children with rheumatic diseases are more susceptible to MTB due to factors such as an immature immune system, the nature of rheumatic diseases themselves, and treatment with anti-rheumatic drugs. LTBI is also more likely to progress to active tuberculosis in these children. From a pathophysiological perspective. After MTB invades the body, pulmonary macrophages are the first to encounter MTB. These macrophages combat MTB through mechanisms such as phagocytosis, lysosomal fusion, recruitment of lysosomal enzymes, production of reactive oxygen species, autophagy, and apoptosis (Mishra et al., 2025). They also upregulate various cytokines, including tumor necrosis factor (TNF)-α, interleukin-1, and granulocyte-macrophage colony-stimulating factor (Gail et al., 2023), to recruit other immune cells to the MTB infection site, thereby helping to control the infection (Ravesloot-Chávez et al., 2021). Dendritic cells that have phagocytosed MTB migrate to peripheral lymph nodes, initiating an adaptive immune response (Chen et al., 2025). They stimulate T lymphocyte differentiation and migration to the MTB infection site, participate in granuloma formation, and produce interferon-γ (IFN-γ), TNF-α, and IL-2 cytokines to eliminate pathogens (Jasenosky et al., 2015). B lymphocytes function as antigen-presenting cells, contributing to the differentiation of MTB-specific T lymphocytes. They also produce MTB-specific antibodies and secrete cytokines that regulate effector cell function, thereby limiting the spread of tuberculosis infection (Rijnink et al., 2021). The host can eliminate MTB infection through both innate and adaptive immune responses. However, MTB can also evade elimination by the immune system through various immune escape mechanisms (Chai et al., 2020). MTB subverts host autophagy through three distinct mechanisms. First, the ESX-1 secretion system perforates the phagosomal membrane, releasing bacterial DNA and RNA. These nucleic acids are detected by cytosolic sensors cGAS-STING and AIM2, which skews the type I interferon response to promote bacterial persistence. Second, MTB rewires host membrane trafficking by retaining Rab5 while excluding Rab7 and Rab20 (Schnettger et al., 2017), and it deploys secreted phosphatases and lipids to prevent phagolysosomal fusion (Buter et al., 2019). Concurrently, MTB diverts canonical apoptotic pathways towards necroptosis or ferroptosis (Amaral et al., 2019). Third, the pathogen blocks autophagic flux by activating mTOR, silencing core autophagy genes via microRNA, and deploying maturation inhibitors. Finally, MTB employs nuclear effectors to epigenetically repress antimicrobial genes (Yaseen et al., 2015), utilizes ubiquitin-modifying enzymes to blunt NF-κB signaling (Wang et al., 2020), and secretes bacterial kinases and phosphatases to disable host signaling pathways and restriction factors (Wang et al., 2015). These coordinated actions cement a long-lived intracellular niche for MTB. LTBI is considered a dynamic balance between MTB and the host immune system.

**Figure 2:**
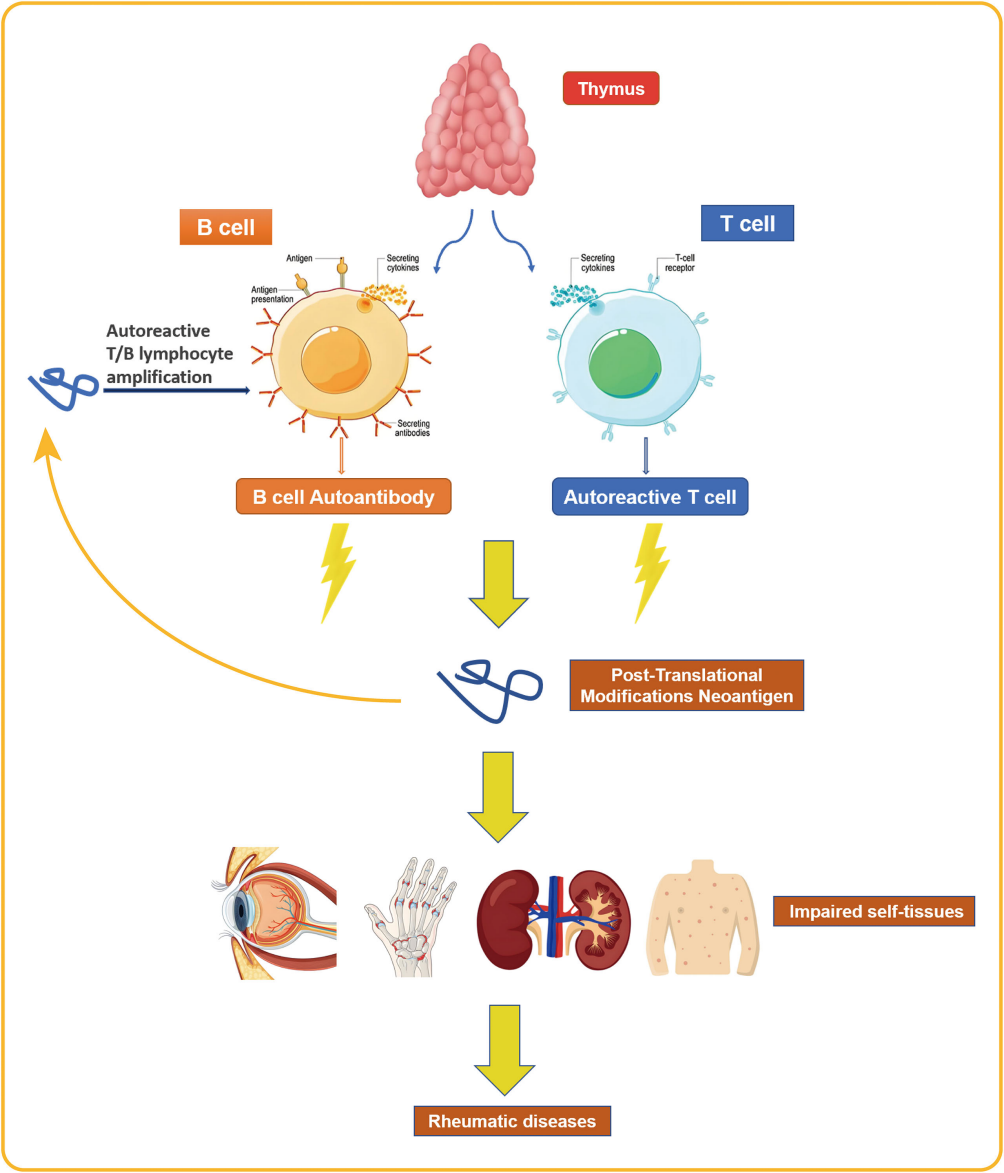
The activation and proliferation of autoreactive T and B cells results in the recognition and damage of the body’s own tissues, a pathogenic process that ultimately leads to the development of rheumatic diseases.

The pathogenesis of rheumatic diseases involves abnormal reactions and activation of T and B lymphocytes (Deng et al., 2024).Incomplete clonal deletion and the presence of neoantigens—such as those generated by post-translational modifications (PTMs)—can activate and expand autoreactive T and B cells. These cells recognize and damage host tissues (**Figure 2**), establishing a state of immune dysregulation and increasing susceptibility to MTB infection (Zhai et al., 2025). Possible mechanisms include T lymphocyte homeostasis imbalance causing T cell exhaustion, resulting in insufficient production of T cells to combat external antigens (Frenz et al., 2016); disrupted T cell function, where naive CD4+ T cells differentiate into effector cells with pro-inflammatory and tissue-invasive properties instead of differentiating into memory T cells involved in infection immunity, thereby weakening the anti-MTB effect (Weyand and Goronzy, 2021); and reduced complement receptor expression, which impairs complement-mediated microbial clearance and increases the risk of infection (Kang and Park, 2003). These immune dysfunctions disrupt the dynamic balance between MTB and the host immune system, leading to the reactivation of LTBI into active tuberculosis and increasing susceptibility to MTB. Existing research indicates that the incidence of LTBI is higher among rheumatic disease patients than in the general population in countries with a high tuberculosis burden (Wang et al., 2022).

The primary medications used in the treatment of rheumatic diseases are various immunosuppressive agents, including corticosteroids and DMARDs. The use of these medications places patients in a temporary state of immunosuppression, which is the primary cause of increased susceptibility to MTB infection and the risk of reactivation of LTBI. The conclusion that corticosteroids increase the risk of MTB infection is relatively clear. Studies have shown that a corticosteroid dose >7.5 mg·d;¹ is an independent risk factor for tuberculosis (Chan et al., 2018), and rheumatic disease patients who receive long-term, daily corticosteroid doses exceeding 15 mg have a significantly increased risk of LTBI reactivation (Long et al., 2020). Patients with rheumatoid arthritis treated with traditional DMARDs have a 3.17-fold higher risk of developing active tuberculosis compared to the general population, with methotrexate, leflunomide, and cyclosporine presenting relatively higher risks (Ai et al., 2015). The introduction of biologic DMARDs has significantly improved the prognosis for children with rheumatic diseases but has also increased their risk of MTB infection, particularly with TNF inhibitors (Gardam et al., 2003). TNF-α inhibitors can suppress the recruitment of T lymphocytes and macrophages to MTB infection sites mediated by TNF-α, hinder granuloma formation, and downregulate the functional activity of macrophages, natural killer cells, and CD8+ T cells. Therefore, patients treated with TNF inhibitors are highly susceptible to progression of LTBI to active tuberculosis (Ravesloot-Chávez et al., 2021). Furthermore, the risk of developing active tuberculosis is higher with TNF-α monoclonal antibodies than with TNF-α receptor antibody fusion proteins (Dixon et al., 2010; Xie et al., 2014). Currently, safety data regarding the use of antirheumatic drugs—particularly biologic and targeted synthetic DMARDs—in pediatric patients with rheumatic diseases remain insufficient. This data gap is especially pronounced in high tuberculosis burden countries. Considering China’s high tuberculosis burden, LTBI screening should be prioritized before and during treatment in children with rheumatic diseases.

### 2.2 Diagnosis of LTBI in children with rheumatic diseases

The use of various anti-rheumatic drugs elevates the risk of both new MTB infection and reactivation of LTBI in children with rheumatic diseases (Li et al., 2022). **Table 1** summarizes the common pharmacologic treatments for juvenile rheumatoid arthritis. Research demonstrates that glucocorticoids suppress key cellular immune responses required to control MTB infection. These suppressive mechanisms include the inhibition of lymphokine activity, impairment of monocyte chemotaxis, and the blockade of Fc receptor function—all of which significantly increase the risk of tuberculosis reactivation in children with rheumatic diseases (Ruscitti et al., 2025). Furthermore, leflunomide, a representative DMARD, promotes immunosuppression by inhibiting pyrimidine synthesis in T and B lymphocytes. Its active metabolite, A77 1726, mediates this effect by blocking the enzyme dihydroorotate dehydrogenase (Tan et al., 2024). This mechanism of action is also associated with an increased risk of tuberculosis reactivation. LTBI diagnostic criteria (Steele et al., 2005; Wang et al., 2022): (1) In individuals who have not received the Bacillus Calmette-Guérin (BCG) vaccine or have no non-tuberculous mycobacteria infection, a mean diameter of ≥5 mm in the purified protein derivative (PPD) test induration indicates the presence of MTB infection; (2) In individuals who have received the BCG vaccine or have NTM infection, a mean diameter of ≥10 mm in the PPD test induration indicates the presence of MTB infection; (3) For children under 5 years of age who have had close contact with active pulmonary tuberculosis patients, or those with human immunodeficiency virus infection or on immunosuppressive therapy for more than 1 month, an average diameter of ≥5 mm in the PPD skin test induration indicates MTB infection; (4) A positive tuberculin skin tests (TST) indicates MTB infection; (5) A positive interferon-gamma release assays (IGRA) test indicates MTB infection.

**Table 1:** Pharmacological management of juvenile idiopathic arthritis.

Drug therapy	Medicines	Dosage	Usage	Maximum dose (mg/d)
NSAIDs	Diclofenac sodium	1-3 mg·kg-1·d-1	Three times daily	150
	Naproxen	10-15 mg·kg^-1^·d^-1^	Twice daily	400
	Ibuprofen	30-40 mg·kg^-1^·d^-1^	Three times daily	1200
	Celecoxib	6-12 mg·kg^-1^·d^-1^	Twice daily	400
Glucocorticoid	Prednisone acetate	1-2 mg·kg^-1^·d^-1^	/	60
	Methylprednisolone	20-30 mg·kg^-1^·d^-1^	/	1000
DMARDs	Hydroxychloroquine	5-6 mg·kg^-1^·d^-1^	/	250
	Sulfasalazine	30-50 mg·kg^-1^·d^-1^	/	/
	Methotrexate	10-15 mg/m^2^	Once weekly	/
	Leflunomide	10-15 mg/d	/	/
	Cyclosporine A	3-5 mg·kg^-1^·d^-1^	/	/
	Cyclophosphamide	300-400 mg/m^2^	Once monthly	/
Biologic Drug	TNF inhibitor	0.4 mg/kg	Twice weekly	/
	IL-6 inhibitor	8-12 mg/kg	Every 2 weeks	/
	Abatacept	10mg/kg	Every 4 weeks	/

The increased use of various antirheumatic drugs has elevated the risk of MTB infection and LTBI reactivation in children with rheumatic diseases. It is recommended that LTBI screening be conducted prior to antirheumatic therapy, with at least one screening per year during treatment (Wang et al., 2022). LTBI individuals exhibit no clinical symptoms or imaging abnormalities, and diagnosis lacks a gold standard. It is primarily assessed through TST or IGRA to detect cell-mediated immune responses to MTB antigens, thereby indirectly evaluating whether infection has occurred (Shah and Dorman, 2021). The TST is the classic method for detecting MTB infection. It involves the subcutaneous injection of tuberculin PPD and assessing the size of the resulting skin induration to determine the result. The TST is simple to perform, cost-effective, and remains an important tool for diagnosing LTBI. However, the PPD used in the TST contains antigens that cross-react with the BCG vaccine and NTM, leading to a high rate of false-positive results and thus reduced specificity (Sun et al., 2011). IGRA determines the presence of MTB infection by measuring the level of IFN-γ released by sensitized T lymphocytes in response to MTB antigen stimulation (Huang et al., 2025). Since the experimental antigens used are not encoded by the genomes of BCG strains and most NTM, such as early secretory antigen-6 and culture filtrate protein-10 (Petruccioli et al., 2016), the results are not influenced by BCG vaccination or most NTM infections, resulting in higher specificity but higher costs (Zhang et al., 2025). Currently, IGRA employs two common detection methods: one is the enzyme-linked immunosorbent assay (ELISA), which measures the level of IFN-γ in whole blood following MTB infection; the other is the T-cell enzyme-linked immunosorbent spot assay (ELISPOT), which uses MTB-specific antigens to stimulate cells and measures the number of T lymphocytes releasing IFN-γ in peripheral blood (Mora-Buch et al., 2025). A meta-analysis evaluating the diagnostic efficacy of these methods in children with tuberculosis infection found that their sensitivity for active tuberculosis was similar (ELISA: 70%, ELISPOT: 62%, TST: 71%). The specificity of ELISA was 100%, ELISPOT: 90%, and TST: 56% (Sun et al., 2011).

A study included 24 children and adolescents with JIA receiving methotrexate treatment (Sztajnbok et al., 2014). The prevalence of LTBI at baseline was 20.8%, the incidence of LTBI after immunosuppressive therapy was 26.3%, and the prevalence of LTBI at the end of the study was 41.6%. The relative risk of developing LTBI among those with a positive tuberculosis epidemiological history was 2.0. Only 2 patients tested positive for T-SPOT.TB, but only in 1 case was the test useful for detecting early LTBI. The sensitivity of T-SPOT.TB was 10%, specificity was 92.8%, and it had low correlation with the TST. Additionally, a comparative study evaluated the performance of the Quantiferon-TB Gold In-Tube (QFT-IT) (IFN)-γ assay in children with LTBI receiving anti-rheumatic therapy (Gabriele et al., 2017). A total of 79 consecutive children receiving anti-rheumatic therapy were tested using both the Mantoux tuberculin skin test (TST) and the QFT-IT. The results suggest that QFT-IT may be a more reliable test than TST for detecting LTBI in children receiving anti-rheumatic therapy. Drug treatment regimens may influence mitogen-induced IFN-γ secretion, and the effects of TNF-α inhibitors may vary depending on the specific drug administered. Due to the lack of a gold standard for LTBI diagnosis, the use of immunosuppressive agents in rheumatic disease patients may result in false-negative TST or IGRA results. In cases where conditions permit, combining TST and IGRA for screening can improve detection sensitivity.

In accordance with the WHO guidelines, either TST or IGRA can be used for LTBI screening. For individuals vaccinated with BCG after infancy or those who have received multiple BCG vaccinations, IGRA is the preferred initial test due to its higher specificity. In high-risk scenarios, such as for immunocompromised patients, a combination of both TST and IGRA may be warranted—particularly prior to initiating TNF-α inhibitor therapy—irrespective of BCG vaccination history (Goletti et al., 2018).

### 2.3 Preventive antituberculosis treatment for LTBI in children with rheumatic diseases

Lima et al. conducted a literature review on TB in children and adolescents with rheumatic diseases who were receiving biologic therapy (Lima et al., 2023), including 81 cases of LTBI, 80 cases of pulmonary tuberculosis, and 4 cases of extrapulmonary tuberculosis. The primary rheumatic disease was JIA. In LTBI cases, most were diagnosed during screening, and none progressed to tuberculosis during follow-up. In tuberculosis cases using biologics, most used anti-TNFα drugs. Only one death occurred.

Children with rheumatic diseases who are found to have LTBI should undergo preventive antituberculosis treatment as early as possible. Studies have shown that children with rheumatic diseases and LTBI who undergo preventive antituberculosis treatment have a good prognosis, with few cases of active tuberculosis or even disseminated tuberculosis. A Spanish cohort study demonstrated that among pediatric patients with rheumatic diseases, preventive treatment for LTBI prior to initiating anti-TNF-α therapy was effective. During a median follow-up period of 6.4 years, active tuberculosis developed in only 1 of the 12 patients diagnosed with LTBI (Calzada-Hernández et al., 2023). However, this patient had been exposed to a tuberculosis patient after LTBI preventive antituberculosis treatment, and the bacterial strains were completely identical, suggesting it was likely a new MTB infection rather than LTBI reactivation. A multicenter study further demonstrated the efficacy of actively administering preventive antituberculosis therapy to children with rheumatic diseases and LTBI, a benefit observed in both high- and low-tuberculosis-burden countries (exemplified by Brazil and Spain, respectively). Among a cohort of 40 children receiving preventive therapy—comprising 31 with LTBI and 9 with a history of tuberculosis exposure—only three subsequently developed active tuberculosis (Piotto et al., 2022).

Currently, there are no pediatric data available regarding the timing of preventive antituberculosis therapy for patients with rheumatic diseases and LTBI. As stipulated in the adult clinical practice guidelines (Wang et al., 2022), treatment strategies must be tailored to disease severity and the urgency of biologic intervention. In cases of severe disease requiring immediate biologic therapy, a thorough assessment of the patient’s risk for LTBI reactivation is imperative; should the risk be deemed significant, biologic agents should be initiated concurrently with preventive antituberculosis treatment. If the condition allows for delaying treatment with corticosteroids, traditional disease-modifying DMARDs, or biologic DMARDs (non-TNF inhibitors), anti-rheumatic therapy should be initiated one month after the completion of preventive antituberculosis therapy. TNF inhibitors should be initiated as late as possible after completion of preventive anti-tuberculosis therapy. According to the expert consensus on the diagnosis and treatment of rheumatic diseases in patients with latent tuberculosis infection in China, there are four main preventive anti-tuberculosis treatment regimens for LTBI in pediatric rheumatic diseases (**Table 2**). The first is the weekly regimen of isoniazid combined with rifapentine (3HP regimen): Take isoniazid plus rifampicin for three months. This regimen is a newly added preventive anti-tuberculosis treatment regimen internationally. Its advantages include a short treatment duration, a high treatment completion rate, and good efficacy. However, its disadvantages include higher costs, although it reduces the number of doses; each dose requires a larger number of tablets (Sterling et al., 2011; Villarino et al., 2015; Sterling et al., 2020). The second is the daily rifampicin monotherapy regimen: taking rifampicin for 4 months. The efficacy is similar to that of isoniazid monotherapy, but with lower hepatotoxicity. The drawback is that it has more drug interactions, such as reduced efficacy of tofacitinib when used in combination with rifampicin (Latent tuberculosis infection: updated and consolidated guidelines for programmatic management, 2018). The third is the daily therapy combining isoniazid and rifampicin: taking isoniazid + rifampicin for 3 months. Compared with the single-drug therapy with isoniazid, the efficacy is similar, but the treatment time is shorter, and the compliance and completion rates are higher. It is one of the recommended first-line treatment regimens internationally (Wang et al., 2020). A significant disadvantage of this combination regimen is the elevated risk of hepatotoxicity compared to monotherapy. A fourth therapeutic option is a six- to nine-month daily course of isoniazid monotherapy. The drawbacks of this approach include an extended treatment duration, a lower treatment completion rate, and a persistent risk of drug-induced hepatotoxicity. Pyridoxine (vitamin B6) supplementation with all isoniazid regimens is recommended to reduce the peripheral neuropathy (Fox et al., 2017).

**Table 2:** Preventive treatment of LTBI in children with rheumatic diseases.

Therapeutic regimen	Medicines	Age 2–14 y				Age >14 y	Maximum dose	Usage	Cycle
		Weight 10-15 kg	Weight 16-23 kg	Weight 24-30 kg	Weight > 31 kg				
Isoniazid + Rifapentine (3HP)	Isoniazid	300mg	500mg	600mg	700mg	900mg	900mg	Once weekly	3 months
	Rifapentine	300mg	450mg	600mg	750mg	900mg	900mg	Once weekly	3 months
Rifampicin monotherapy	Rifampicin	10mg/kg body weight per dose					450mg	Once daily	4 months
Isoniazid + Rifampicin	Isoniazid Rifampicin	10mg/kg body weight per dose					300mg 450mg	Once daily	3 months
Isoniazid monotherapy	Isoniazid	10mg/kg body weight per dose					300mg	Once daily	6-9 months

Preventing the progression of drug-resistant tuberculosis (DR-TB) infection to active disease is a critical pillar of the global DR-TB elimination strategy. International guidelines have recently proposed fluoroquinolones for tuberculosis preventive therapy in DR-TB contact. The pooled data from small observational studies suggest that fluoroquinolone-based TPT is safe, effective, and cost-effective for this purpose (Kherabi et al., 2022).For children with rheumatic diseases exposed to a household case of multidrug-resistant tuberculosis, prevention must commence with a personalized risk assessment. In February 2024, the WHO issued rapid guidance endorsing a once-daily, six-to-nine-month regimen of levofloxacin, which significantly reduces the risk of progression from latent infection to active disease in this vulnerable population (Seddon et al., 2018). Additionally, the M72/AS01E vaccine has demonstrated 50% efficacy in preventing this progression, presenting a promising future intervention (Latent tuberculosis infection: updated and consolidated guidelines for programmatic management, 2018).

## 3 Conclusion

The incidence of LTBI in patients with rheumatic diseases is rising. Children are particularly vulnerable to MTB infection and progression to active tuberculosis due to immune dysregulation, inherent disease factors, and immunosuppressive therapy. Prioritizing standardized LTBI screening, timely preventive treatment, and effective intervention is crucial to reducing active tuberculosis incidence. Current global guidelines for LTBI management in this population—covering risk stratification, screening, and prevention—remain inadequate. Strengthening collaboration between tuberculosis and rheumatology disciplines to advance high-quality clinical research is urgently needed to inform evidence-based practice.
